# 1629. Acceptability and Trust in the Low-dose Intradermal Mpox Vaccine: a Knowledge, Attitudes and Practices Sub-analysis of the Observational Study of Mpox Immunity (OSMI) in NYC

**DOI:** 10.1093/ofid/ofad500.1463

**Published:** 2023-11-27

**Authors:** Ellie Carmody, Lalitha Parameswaran, Kesi K Wilson, Angelica C Kottkamp, Olivia Frank, Celia Engelson, Tamia S Davis, Irma Noriega, Jacqueline Callahan, Samantha Yip, Heekoung Youn, Stephanie Rettig, Julia Wagner, Samuel Nweke, Pamela Suman, Marie I Samanovic, Ralf Duerr, Sarah Haiken, Mark J Mulligan

**Affiliations:** NYU Langone Health, BROOKLYN, New York; NYU Langone Health, BROOKLYN, New York; NYU Grossman School of Medicine, Brooklyn, New York; NYU Grossman School of Medicine, Brooklyn, New York; NYU Langone, new york, New York; NYU Vaccine Research Center, New York, New York; NYU Langone Health Vaccine Center, Yonkers, New York; NYU Langone Medical Center, NY, New York; NYU Langone Health Vaccine Center, Yonkers, New York; NYU Grossman School of Medicine, Brooklyn, New York; NYU Langone Health, BROOKLYN, New York; NYU Langone Vaccine Center, New York, New York; NYU Grossman School of Medicine, Brooklyn, New York; NYU Langone Health, BROOKLYN, New York; NYU Langone, new york, New York; NYU Grossman School of Medicine, Brooklyn, New York; NYU Langone Health, BROOKLYN, New York; NYU Langone Health, BROOKLYN, New York; NYU Grossman School of Medicine, Brooklyn, New York

## Abstract

**Background:**

In the 2022 Mpox outbreak, the US FDA issued an EUA for a low-dose intradermal (ID) vaccination regimen to increase vaccine supply by five-fold. The ID regimen had yet to be evaluated for immunogenicity or tolerability in persons with HIV or for general acceptability.

**Methods:**

We conducted a Knowledge, Attitudes and Practices survey to evaluate ID Mpox vaccine acceptability, comparing responses between participants with and without HIV and by route received.

**Results:**

Of 119 ethnically and racially diverse respondents, 83% identified as male, 91% as LGBTQ+, median age was 38 years. 26% had HIV. 21% received ID/ID, 57% mixed subcutaneous (SC)/ID or ID/SC, and 18% SC/SC regimens. Vaccination route was similarly distributed across age, race, ethnicity, educational status, household income and HIV status. Knowledge of Mpox vaccines was moderate, with a mean of 4.36 of 7 responses correct. 62% were satisfied with Mpox vaccine rollout and 20% were neutral. 77% agreed that they trusted information received about the Mpox ID route by healthcare providers. Respondents with HIV were less likely to fully support the policy change to the ID route to stretch vaccine supply (Mann-Whitney p=0.039), but otherwise there were no significant differences between respondents with and without HIV in satisfaction with Mpox vaccine rollout, trust in vaccine-related information, confidence about ID regimen’s safety, efficacy, concern about cosmetic side effects, or overall acceptability. There were no significant differences between respondents by vaccination regimen in satisfaction with vaccine rollout, approval of the change toward ID formulation, concern about cosmetic side effects or overall acceptability. Compared to those who received mixed or ID/ID dosing regimens, SC/SC participants exhibited less confidence in efficacy of the ID formulation (Kruskal-Wallis p=0.015) and lower trust in information received by the US government about the ID formulation (p=0.01). Overall, 88% scored their Mpox vaccination experience as “excellent or very good,” and there was no difference between groups.

**Figure:**
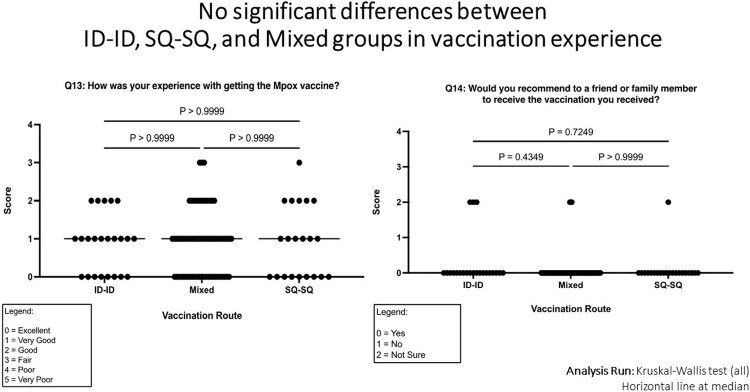

**Figure:**
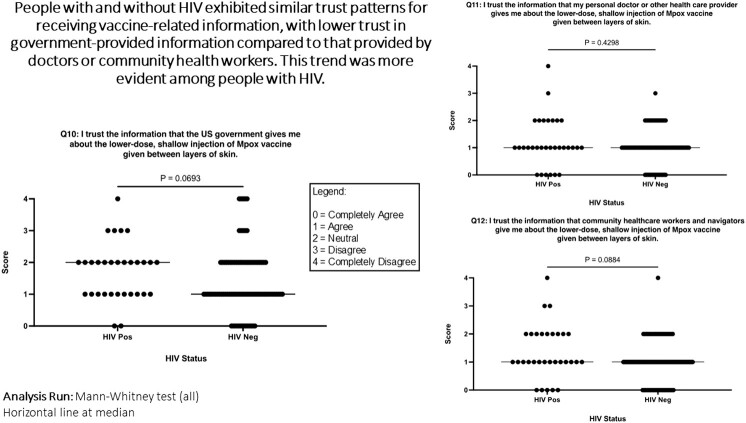

**Conclusion:**

Survey responses from this clinical study sample suggest high acceptability of the ID regimen for Mpox vaccination in all groups, including among people with HIV and those who had received this regimen.

**Disclosures:**

**Ellie Carmody, MD, MPH**, AstraZeneca: Stocks/Bonds|Merck: Stocks/Bonds **Lalitha Parameswaran, MD**, Pfizer: Grant/Research Support **Mark J. Mulligan, M.D.**, Lilly: Grant/Research Support|Meissa Vaccines, Inc.: Advisor/Consultant|Meissa Vaccines, Inc.: Board Member|Merck: Advisor/Consultant|Merck: Board Member|Pfizer: Advisor/Consultant|Pfizer: Board Member|Pfizer: Grant/Research Support|Sanofi: Grant/Research Support

